# Mongolian Almond (*Prunus mongolica* Maxim): The Morpho-Physiological, Biochemical and Transcriptomic Response to Drought Stress

**DOI:** 10.1371/journal.pone.0124442

**Published:** 2015-04-20

**Authors:** Jǖgang Wang, Rong Zheng, Shulan Bai, Xiaomin Gao, Min Liu, Wei Yan

**Affiliations:** 1 College of Forestry, Inner Mongolia Agricultural University, Hohhot, Inner Mongolia, China; 2 College of Life Science and Technology, Inner Mongolia Normal University, Hohhot, Inner Mongolia, China; 3 College of Agriculture, Inner Mongolia Agricultural University, Hohhot, Inner Mongolia, China; 4 Institute of Forestry Science of Ordos, Ordos, Inner Mongolia, China; Chinese Academy of Sciences, CHINA

## Abstract

*Prunus mongolica* Maxim, which is widely established in the Gobi Desert, shows extreme tolerance to drought. However, there is a lack of available transcriptomic resources for this species related to its response to water deficiency. To investigate the mechanisms that allow *P*. *mongolica* to maintain growth in extremely arid environments, the response of *P*. *mongolica* seedlings to drought stress was analyzed using morphological, physiological, biochemical and high-throughput sequencing approaches. We generated 28,713,735 and 26,650,133 raw reads from no-stress control and drought-stressed *P*. *mongolica* seedlings, respectively. In total, we obtained 67,352 transcripts with an average length of 874.44 bp. Compared with the no-stress control, 3,365 transcripts were differentially expressed in the drought-stressed seedlings, including 55.75% (1,876 transcripts) up-regulated and 44.25% (1,489 transcripts) down-regulated transcripts. The photosynthesis response showed a decreasing tendency under drought stress, but the changes in the levels of hormones (auxins, cytokinins and abscisic acid) resulted in the closing of stomata and decreased cell enlargement and division; these changes were effective for promoting *P*. *mongolica* survival in Gobi Desert. Next, we analyzed the aquaporin and superoxide dismutase gene families due to their importance in plant resistance to drought stress. We found that all of the plasma membrane intrinsic protein transcripts were down-regulated in the drought-stressed treatment, whereas drought did not affect the expression of nodulin intrinsic protein or small basic intrinsic protein transcripts in *P*. *mongolica* seedlings. In addition, activation of iron superoxide dismutase transcription and enhanced transcription of manganese superoxide dismutase were observed in *P*. *mongolica* to promote tolerance of drought stress. This study identified drought response genes in *P*. *mongolica* seedlings. Our results provide a significant contribution to the understanding of how *P*. *mongolica* responds to drought stress at the transcriptome level, which may help to elucidate molecular mechanisms associated with the drought response of almond plants.

## Introduction

Most terrestrial ecosystems in nature are rain fed, and drought poses a major limitation to productivity in these ecosystems. Recurrent dry periods and scattered rainfall patterns have resulted in water shortages and the consequent loss of or damage to crop production in sufficiently rain-fed areas [1]. In addition, increased atmospheric CO_2_ concentrations may increase in the severity of drought conditions of some arid and semi-arid regions [2]. Atmospheric CO_2_ concentrations rose from 280 to 368 ppm during the 20th century and may rise to > 700 ppm by the end of the 21st century (IPCC, 2007). Elevated CO_2_ concentration leads to the increase of global mean temperature, reduces the rainfall and increases the evaporation especially in arid and semi-arid regions [3,4]. According to a recent estimation, the economic losses caused by drought may be as high as $80 billion per year worldwide, which may be due not only to the lack of water causing a decreased yield potential but also to the timing and duration of drought stress events in relation to plant phenological processes [5]. Droughts are expected to continue becoming longer and more severe in some regions. However, high relative yields of certain genotypes have been documented following exposure to drought stress [6]. Thus, it is important to reveal the unique molecular and biochemical mechanisms associated with drought tolerance in some extreme plants, such as desert plants.

**Fig 1:**
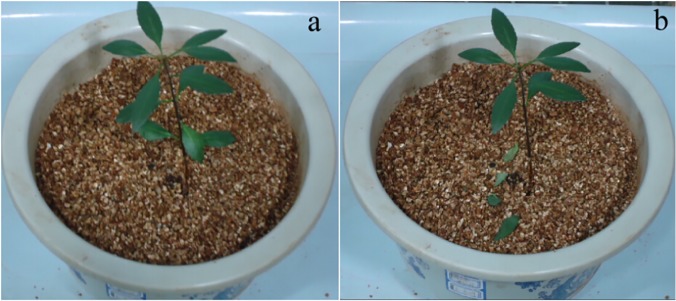
*P*. *mongolica* seedling, before (a) and after (b) the drought-stressed treatment.

Mongolian almond (*Prunus mongolica* Maxim, Fig 1) is distributed widely in the Gobi Desert of the Mongolian plateau. This species is an ancient relict flora that has been recorded as a rare plant on the China Plant Red List and adopted as a state key conservation species [7]. While *P*. *mongolica* grows well in water- and nutrient-limited desert areas and plays an important role in the local economy and ecological environment, no studies have been reported investigating the mechanisms (particularly the molecular mechanisms) underlying the drought tolerance of this shrub.

The drought stress response in plants is followed by morphological and biochemical changes, such as a deeper root system, closure of stomata, shedding of older leaves [8], strengthening of reactive oxygen species (ROS) scavenging mechanisms and increases in the accumulation of osmoregulation substances [9]. These changes are effective for allowing survival under drought conditions. The mechanisms underlying the response to drought stress can be measured at many different levels, from the whole plant to the molecular level. Because stress responses are controlled by the plant genome, recent efforts have focused on the molecular response of the plants to drought stress [10]. The development of next-generation, high-throughput sequencing technologies has provided a significant and low-cost way to generate genome/transcriptome resources for non-model organisms at large scales [11]. Transcriptome research conducted in *Agave deserti* [12], *Boehmeria nivea* [13], *Chenopodium quinoa* [14], *Manihot esculenta* [8], *Paulownia australis* [15], *Populus euphratica* [16] and *Pseudotsuga menziesii* [17] has been used to successfully identify differentially expressed genes, implicating several biosynthetic pathways that assist in the overall tolerance to drought stress. For *Prunus* plants, Alimohammadi et al (2013) [18] identified the water-deficit resistance genes in wild almond (*P*. *scoparia*) using cDNA-AFLP technology, the drought stress-responsive miRNAs in peach (*P*. *persica*) [19] and the freezing stress-responsive genes in *P*. *dulcis* [20] were analyzed by the high-throughput sequencing technology.

In this study, we characterized the seedling transcriptome of *P*. *mongolica* during the response to drought stress. A core set of stress-related transcripts were determined, together with morphological changes (growth status), physiological process and biochemical functions, to provide a comprehensive analysis of drought acclimation in this species. The acquired information may provide new insights into the molecular mechanisms underlying *P*. *mongolica*’s response to drought stress.

## Materials and Methods

### Seedling cultivation and water stress treatments

The original seeds used in this study were collected from the wild *P*. *mongolica* which distributed in the Tengger Desert, Inner Mongolia, Northwest China (103°20′ E, 39°14′ N) on August 10, 2012. After their endocarp being removed by physical damage, the seeds were surface-sterilized with 10% H_2_O_2_ for 30 min and washed with sterilized water. Then they were germinated at 27°C in Petri dishes (diameter = 9 cm). The germinated seeds were transplanted into plastic pots (1 seedling per pot, cylindrical pots: 12×8×15 cm) containing 550 g of sterilized vermiculite (121°C, 90 min) and watered weekly with 150 mL of Hoagland’s solution for 45 days before being subjected to stress treatment in an experimental greenhouse under controlled environmental conditions (irradiation: 16.8 klx; day/night cycle: 14/10 h; temperature: 25/20°C).

In total, 72 uniform 45-day-old *P*. *mongolica* seedlings, including 36 controls and 36 water-deprived plants, were used to determine all parameters nondestructively throughout the experiment. The controls (WW) were watered daily to field capacity, with a 48.7% soil volumetric water content being recorded (measured using a Field Scout TDR 300 Soil Moisture Meter, Spectrum Technologies, USA). The drought stress treatment (DS) was implemented to simulate a gradual soil drying process, which was similar to a natural drought event. This treatment began on March 10, 2013 and was extended for 15 days without irrigation. The soil volumetric water content was 6.3% after drought stress.

All of the *P*. *mongolica* seedlings were harvested after being cultured in the greenhouse for a total of 60 days. Twelve seedlings from each treatment were thoroughly washed with sterile water. Rapid freezing was performed by submerging the samples in liquid nitrogen, followed by storage at -80°C until RNA extraction and high-throughput sequencing. The other 24 seedlings from each treatment were collected to assess growth and physiological and biochemical parameters as described below.

### Growth, physiological and biochemical measurements

To evaluate the growth status of *P*. *mongolica* seedlings under drought conditions, growth-related parameters were determined in 24 seedlings from each treatment at the initial stage of water stress and at the final harvesting time, including the leaf number, length and width. After drought stress, the contents of auxin, abscisic acid and cytokinins were quantified via enzyme-linked immunosorbent assay (ELISA) [21] according to the manufacturer’s protocol, and total superoxide dismutase (SOD) (EC 1.15.1.1) activity was assessed according to the method described by Alvarez et al. (2009) [22].

All physiological measurements were performed daily at 11:00 during the process of drought stress. The net photosynthetic rate (Pn), stomatal conductance (Gs) and leaf water potential (LWP) were determined to identify the physiological adjustment of *P*. *mongolica* to the water stress treatment. Gs and Pn were measured using a LICOR-6400 portable photosynthesis system (LI-COR Inc., USA). The LWP (ψ_leaf_) was measured on the abaxial leaf surface in intact plants using a PSYPRO Water Potential System (WESCOR Inc., USA).

All data obtained from the growth, physiological and biochemical measurements were subjected to one-way ANOVA at each harvesting time using SAS software (SAS Institute Inc., USA). The different treatment types were compared via Duncan’s multiple range tests at a 5% significance level (n = 12).

### RNA extraction, mRNA library construction and sequencing

Total RNA was prepared using TRIzol (Invitrogen) from a mixture of 12 seedlings from each treatment. RNA integrity was analyzed using an Agilent bioanalyzer (Agilent Technologies 2000). mRNAs were purified with oligo (dT) magnetic beads, then fragmented and used to synthesize cDNA following the TruSeq RNA Sample Preparation v2 Guide (Illumina). Sequencing adaptors were ligated to cDNA fragments through PCR amplification. Raw sequencing data were generated using the Illumina HiSeq 2000 system (Illumina, USA) and the sequencing procedure was performed by CapitalBio Corporation (China). The raw data presented in this publication were deposited in the NCBI Short Read Archive (http://www.ncbi.nlm.nih.gov/sra/) and are accessible using the following SRA accession number: SRP049799 (experiment accession numbers SRX759607 for the DS treatment and SRX759609 for the WW treatment).

### Processing and assembly of sequencing reads

The raw data were processed before assembly. Low-quality reads were excluded according to their compliance with the following standards: (1) from the beginning base of each read, the quality value of each base cannot be less than 10—this base and the following bases were removed if the quality value was less than 10; and (2) the length of reads (2 reads of paired-end sequencing) must be greater than 30 bp after filtering. A *de novo* assembly method [23] was applied due to the lack of available genome information for *P*. *mongolica*. The obtained clean reads (Q20, calculate by CASAVA 1.8.2) were assembled with assembly software (trinityrnaseq-r2013-02-25) to construct unique consensus sequences [24]. The trimmed Solexa transcriptome reads were mapped onto the unique consensus sequences using Bowtie2 (Bowtie parameter: –v3—all—best—strata) [25]. A Perl script was written to process the mapping results and generate transcripts.

### Functional annotation and classification

The obtained transcripts were compared with the NCBI non-redundant nucleotide database (NT, by Jan 2013) and the non-redundant protein database (NR, Nov, 2014) using BLASTN [26] and BLASTX [26], respectively, with the same E-value cutoff of ≤e^-5^. Transcripts were identified based on comparison of sequence similarity against SWISS-PROT (downloaded from the European Bioinformatics Institute: ftp://ftp.ebi.ac.uk/pub/databases/swissprot/ by Jan, 2013) using BLAST [26] at E-values ≤e^-10^. Transcripts were assigned functional annotation through comparison of sequence similarity against the Clusters of Orthologous Groups of proteins database (COG, http://www.ncbi.nlm.nih.gov/COG/) [27,28] with BLAST [26] at E-values ≤e^-10^, and a Perl script was written to assign functional classes to the transcripts. The transcripts were first compared with the Kyoto Encyclopedia of Genes and Genomes database (KEGG, http://www.genome.jp/kegg/, release 58) [29] using BLASTX [26] at E- values ≤e^-10^. A Perl script was employed to retrieve KEGG Orthology (KO) information from the BLAST results and subsequently establish pathway associations between the transcripts and database. InterPro domains [30] were annotated with InterProScan [31] Release 27.0, and functional assignments were mapped onto the gene ontology (GO, http://beta.geneontology.org) [32]; WEGO (http://wego.genomics.org.cn/) [33] was used for GO classification and to draw the GO tree.

### Detection of differentially expressed transcripts

Similar to the credibility interval approaches reported for the analysis of SAGE [34] data, we employed IDEG6 [35] to identify transcripts showing statistically significant differences in relative abundance (gene expression levels were calculated using the transcript read number per million (TPM) method [36]) between the two libraries. The ratio was used to determine the differentially expressed transcripts. Transcripts exhibiting ratio values above 2.0 were regarded as up-regulated transcripts, while transcripts presenting values below 0.5 were regarded as down-regulated transcripts [16].

$$\text{ratio}=\frac{\text{transcript TPM(DS)}+1}{\text{transcript TPM(WW)}+1}$$ (1)

Note: 1 is added to the TPM value to prevent the inability to perform the calculations when the TPM value is equal to 0.

### Quantitative real-time PCR

Quantitative real-time PCR (qRT-PCR) was performed to validate the results obtained from high-throughput sequencing. New RNA was isolated using TRIzol (Invitrogen) from a mixture of 12 seedlings from each treatment, purified with absolute alcohol and treated with DNase (EN0521, Thermo). The RNA was reverse-transcribed using the RevertAid First-Strand cDNA synthesis Kit (K1621, Thermo) following the manufacturer’s instructions. Quantitative reverse transcription PCR (qRT-PCR) was performed using the LightCycler 480 real-time PCR system (Roche). The reaction mixture (25 μL) contained 2× Maxima SYBR Green qPCR Master Mix (12.5 μL), 1 μM each of the forward and reverse primers (1 μL, the primers are listed in S1 Table), 2 μL of template cDNA and 9.5 μL of nuclease-free water. PCR amplification was conducted under the following conditions: 95°C for 10 min, followed by 40 cycles at 95°C for 15 s and 60°C for 60 s. Three independent biological replicates for each sample and three technical replicates of each biological replicate were analyzed in the quantitative real-time PCR analysis. The gene expression of the selected transcripts was normalized against an internal reference gene, glyceraldehyde-3-phosphate dehydrogenase [37] (GAPDH) (comp67082_c0_seq1). Relative transcript expression was calculated using the 2^-ΔΔCt^ method [38].

## Results and Discussion

### RNA-seq of *P*. *mongolica* transcriptome

**Fig 2:**
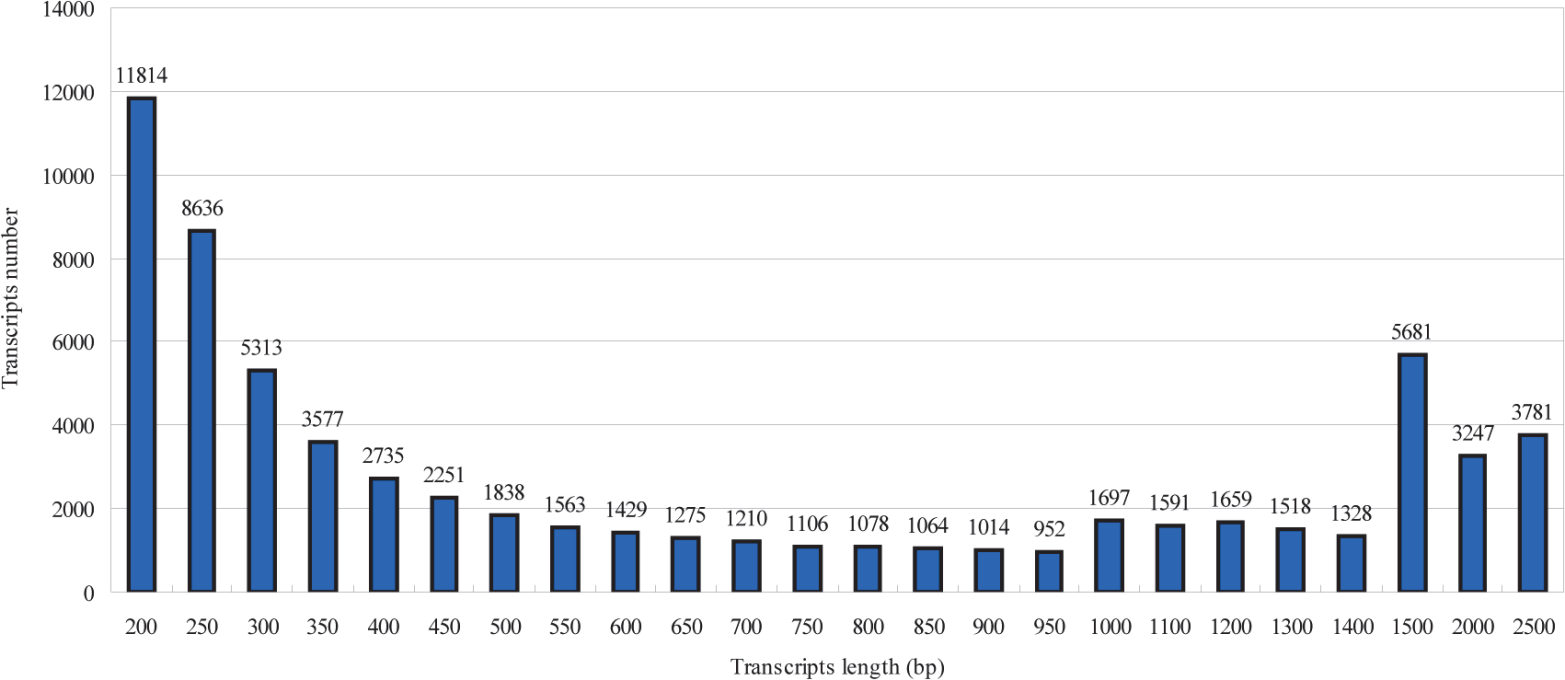
Size distribution of the total *P*. *mongolica* transcripts.

A total of 28,713,735 and 26,650,133 raw reads were generated from the WW and DS *P*. *mongolica* seedlings, respectively. After removing low-quality reads, a total of 26,851,249 clean reads were obtained for the WW treatment and 24,826,653 clean reads were obtained for the DS treatment. To generate more complete and representative information about the *P*. *mongolica* transcriptome, all of the clean reads from both libraries were mapped to transcripts. A total of 67,352 transcripts were obtained, ranging from 0.2 to 2.5 kb, with an average length 874.44 bp (Fig 2). Compared with previous studies [11,16,17] conducted using the 454 platform, both our results and the results of other studies [39,40] employing the same Solexa platforms show that Solexa sequencing technology can generate more clean reads mapped to transcripts and can be widely applied in non-model plant transcriptome sequencing. In addition, 63,613 transcripts were assembled in the DS library, which was a greater number than in the WW library, with 62,107 transcripts. Our results were consistent with previous conclusions indicating an activated transcriptome in plants in response to drought stress [17,41].

### General features of the transcriptome of *P*. *mongolica*

**Fig 3:**
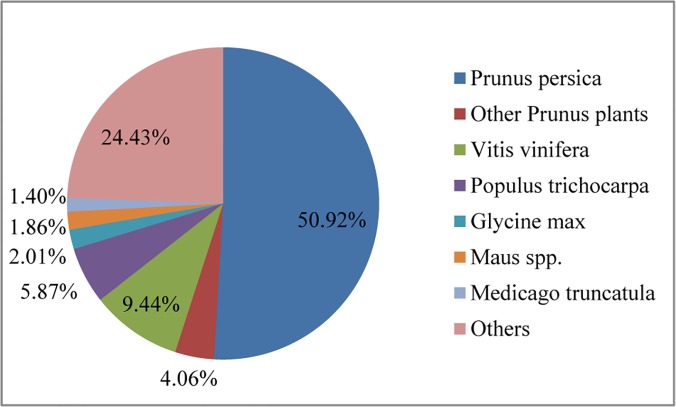
The distribution of the top BLAST hits for the total *P*. *mongolica* transcripts.

The general features of the transcriptome of *P*. *mongolica* may yield potential information to understand the drought adaption of this shrub. The results for the 67,352 transcripts when matched with 7 databases are presented in Table 1. In total, 55,126 (81.8%) transcripts showed matches with the Nr database, 32,716 (48.6%) with the SwissProt database, and 29,719 (44.1%) with the InterPro database. Of the 55,126 transcripts displaying the best hits in the Nr database, 41,656 (75.6%) transcripts corresponded to known plant’s protein sequences, with 28,069, 2,236, 5,205, 3,238, 1,109, 1,027 and 772 transcripts matching sequences from *P*. *persica*, other *Prunus* plants (*P*. *nume*, *P*. *dulcis* and *P*. *avium*), *Vitis vinifera*, *Populus trichocarpa*, *Glycine max*, *Malus* spp. and *Medicago truncatula*, respectively (Fig 3). This result indicated that *P*. *mongolica*’s transcripts have a high level (50.9%) of annotation similarity with peach. When adding the same genus plants, including *P*. *nume*, *P*. *dulcis* and *P*. *avium*, this level will arise to 55.0%. In addition, we note that only 28,927 protein annotations appeared in the high quality draft genome of peach [42], but 28,069 transcripts properly hits peach protein sequence, accounted for 97.0% of peach protein sequences has been annotated. This could show high accuracy of our assembly and could reflect the presence of high similarity between Mongolian almond and peach genomes. The high level of homology between peach and almond plants as well as other *Prunus* genomes has been also reported in the previous reports [20,43,44]. These similarities could indicate that the quality of our assembly is good enough to proceed to the next steps of analysis.

**Table 1:** Summary of *P*. *mongolica* transcripts matched with the Nt (non-redundant nucleotide), Nr (non-redundant protein), SwissProt, COG (Clusters of Orthologous Groups of proteins), KEGG (Kyoto Encyclopedia of Genes and Genomes), InterPro and GO (Gene Ontology) databases.

*Database*	*Transcript annotation No*. *(Percentage)*	*E cut off*	*Database version*
Nt	47320 (70.3%)	1.00×e^-5^	201301
Nr	55126 (81.8%)	1.00×e^-5^	201411
SwissProt	32716 (48.6%)	1.00×e^-10^	201301
COG	21176 (31.4%)	1.00×e^-10^	No version
KEGG	47306 (70.2%)	1.00×e^-10^	Release 58
InterPro	29719 (44.1%)	—	InterProScan 4.8
GO	24553 (36.5%)	—	V36
Total	67357 (100%)	—	—

For an overview of the transcripts that matched the COG database, see S2 Table. Detailed information, including the protein name in COG, BLAST E-value, function ID, COG ID, COG class definition and functional categories (first level and second level), is provided in S3 Table. Among the 21,176 transcripts, nearly one-third were annotated as function unknown or had only a general function prediction. Proteins with uncharacterized functions form a large part of many of the currently available biological databases [45], and this situation even exists in model plant species. The function of the proteins encoded by approximately 13% of the *Arabidopsis thaliana* genome is classified as completely unknown, and the functions of >30% of *Arabidopsis* proteins are poorly characterized [46] (http://www.arabidopsis.org/). However, many of these genes of unknown function have been demonstrated to play a key role in the response of plants to abiotic stresses [47]. The transcripts of unknown function found in *P*. *mongolica* might also play an important role in the response of *P*. *mongolica* to drought stress, and we will aim to elucidate the roles of these uncharacterized transcripts in our future research.

**Fig 4:**
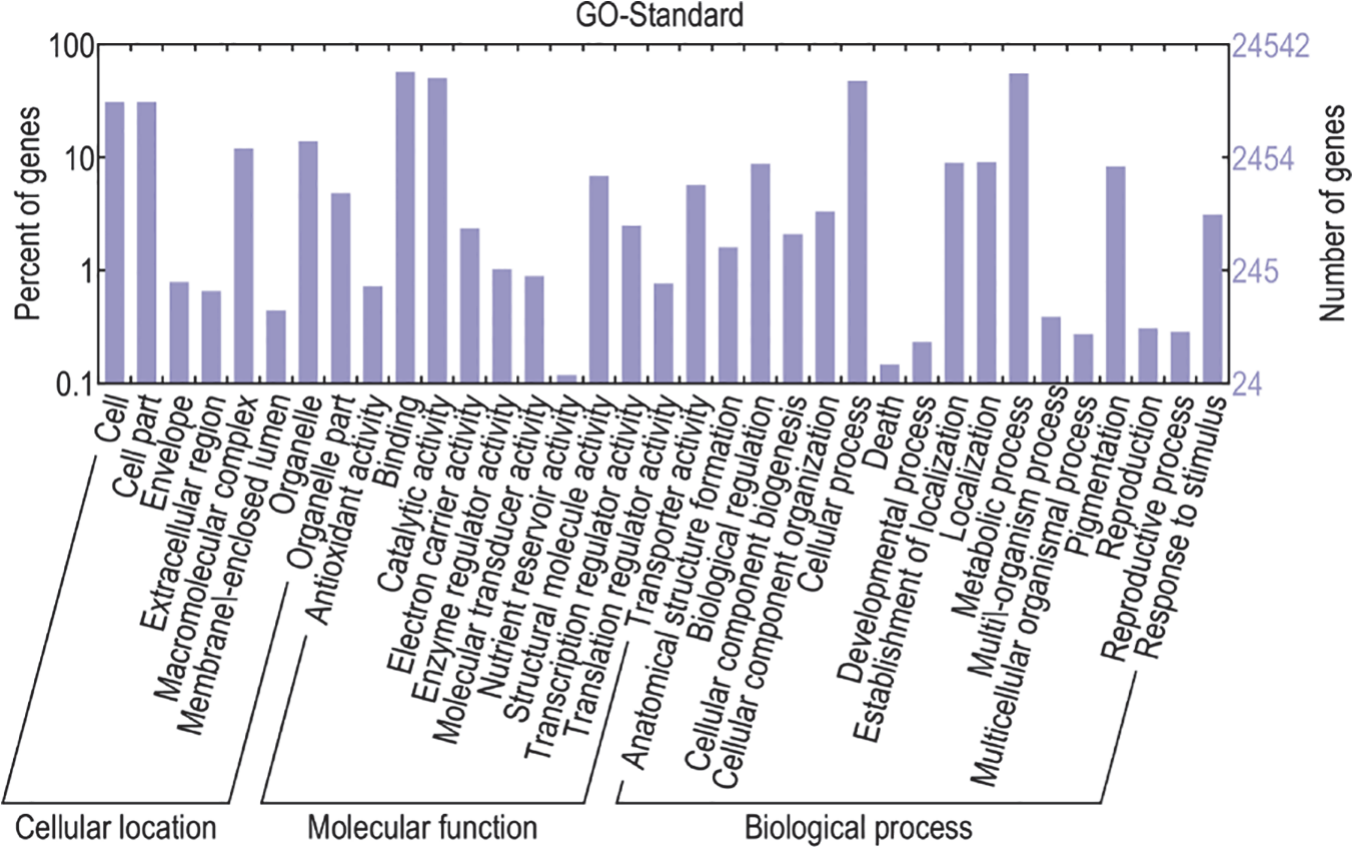
GO classification of the total *P*. *mongolica* transcripts.

All of the transcripts were assigned a GO classification (Fig 4). In total, 31,357 transcripts significantly matched GO terms in the molecular function category; 23,093 in the cellular component category; and 36,684 in the biological process category. Among standard molecular functions, 12 types of functions were covered in our data. Among these functions, binding (13,917) and catalytic activity (12,324) were the most dominant categories. This result coincides with the previous conclusion that binding and catalytic activity operate universally at the transcriptome level [16,48]. Pathway-based analysis helps to further elucidate the roles that genes play in different biological functions. KEGG can assign different transcripts to different biochemical pathways using EC (enzyme commission) members. Overall, this analysis revealed the transcripts showed similarities to sequences in the KEGG database and assigned the transcripts to 232 different pathways (see S4 Table).

### General analysis of differentially expressed transcripts

To identify transcripts that are critical in the drought stress response, we analyzed the differentially expressed transcripts (DETs) between the DS and WW treatments. At a significance level of 0.01 (P value) and an absolute Log_2_(ratio) ≥1, we identified 3,365 reliable DETs, including 1,876 up-regulated transcripts and 1,489 down-regulated transcripts, in the DS treatment compared with the WW treatment.

**Fig 5:**
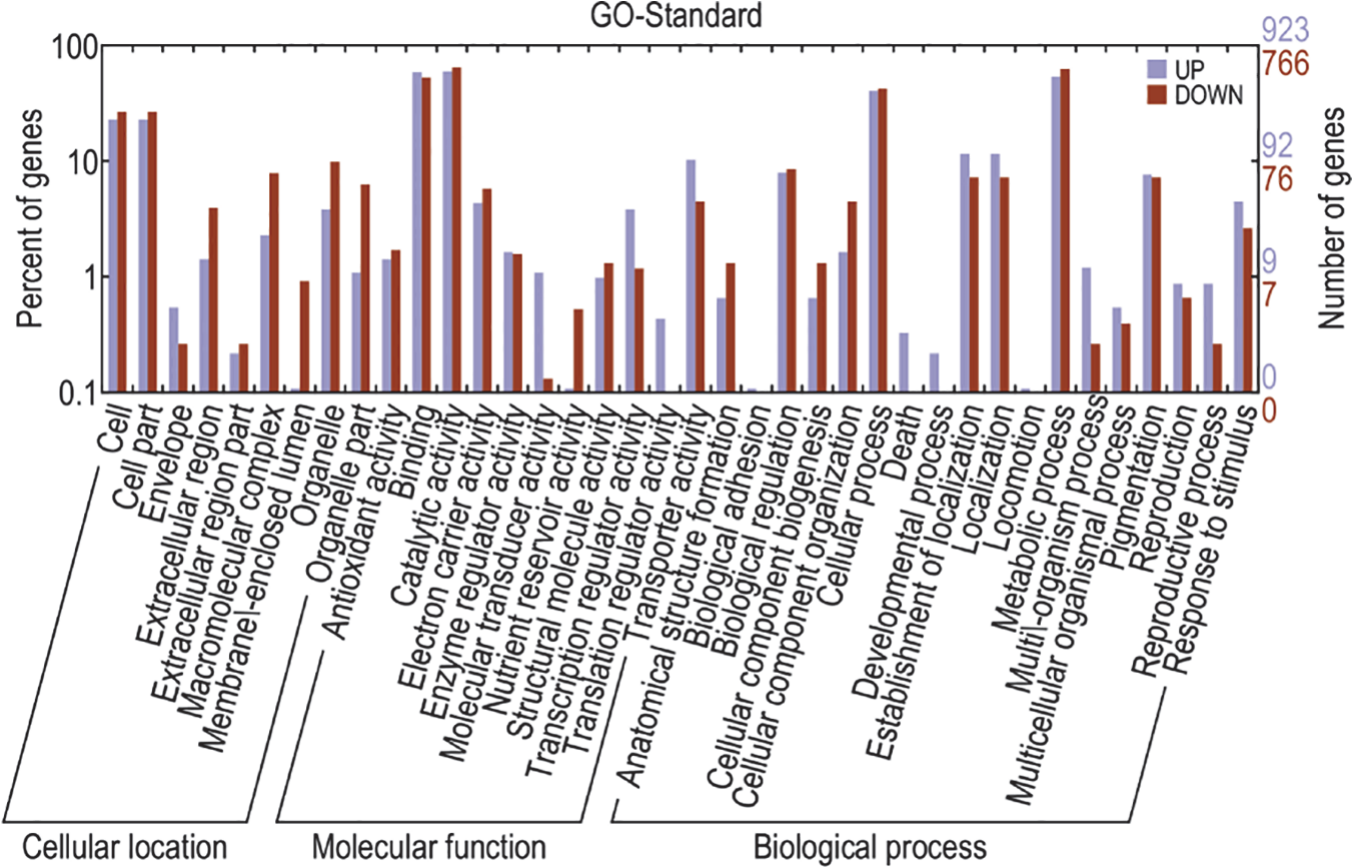
GO classification of the DETs found in *P*. *mongolica*.

Based on the GO annotations obtained using WEGO, the 3,365 reliable DETs were subjected to functional enrichment analysis. The GO functional categories of the up- and down-regulated transcripts, including biological processes, molecular functions and cellular components, are presented in Fig 5. The GO classification results showed that the up-regulated transcripts were mainly involved in the binding, catalytic activity, cellular process, metabolic process, molecular transducer activity, translation regulator activity, envelope, death and developmental process categories, whereas the exclusively down-regulated transcripts displayed functions in the binding, catalytic activity, cellular process, metabolic process, membrane-enclosed lumen, nutrient reservoir activity and macromolecular complex categories.

The bottom leaves of the DS seedlings became brown and curled up, and 3.33 older leaves were shed per seedling on average (see Table 2) under drought stress. In the sequencing data, up-regulated transcripts with GO terms involved in death and developmental processes only appeared under the DS treatment. These two results indicate that a portion of the plant cells were dying and that the plants accelerated the developmental process to address drought stress [49] after an extended period without irrigation. Our findings were consistent with studies on *Populus simonii* [50] and *Camellia sinensis* [51].

**Table 2:** The growth status of well-watered (WW) and drought-stressed (DS) *P*. *mongolica* seedlings on March 10^th^ and March 25^th^, 2013.

*Treatment*	*Leaf length (cm*, *3/10/2013)*	*Leaf width (cm*, *3/10/2013)*	*Leaf length (cm*, *3/25/2013)*	*Leaf width (cm*, *3/25/2013)*	*Number of leaves shed*
WW	3.28±0.16a	1.31±0.08a	3.78±0.23a	1.52±0.21ab	0.17±0.16b
DS	3.26±0.22a	1.33±0.15a	3.37±0.19b	1.37±0.16b	3.33±1.33a

Different letters in the same column indicate a significant difference between the WW and DS treatments: Duncan’s multiple range tests at the 5% level (n = 12). The presented data are the means of 12 replicates, and the standard errors of the means are shown.

The DETs were also matched with KEGG, and the top 20 KEGG pathways are shown in Table 3. Various significant pathways were observed to be related to *P*. *mongolica* drought tolerance, involving transcription factors, plant hormone signal transduction, starch and sucrose metabolism and cysteine and methionine metabolism. Transcription factors are major players in drought stress signaling, and some constitute major hubs in these signaling webs [52]. For adaptation to an environment showing a water deficit, transcription factors regulate the expression of special genes [53], and the zinc finger structure of transcription factors is composed of a polypeptide chain enriched in cysteine [54]. Thus, cysteine and methionine metabolism were increased in the *P*. *mongolica*’s transcriptome.

**Table 3:** Top 20 KEGG pathways of differentially expressed transcripts (DETs).

KEGG pathway	A (B)	Percentage (%)	Up/Down
ko00194: Metabolism; Energy metabolism; Photosynthesis proteins	168 (35)	20.8	1/34
ko04110: Cellular processes; Cell growth and death; Cell cycle	224 (32)	14.3	8/24
ko03032: Genetic information processing; Replication and repair; DNA replication proteins	315 (29)	9.2	6/23
ko03036: Genetic information processing; Replication and repair; Chromosome	711 (27)	3.8	8/19
ko03000: Genetic information processing; Transcription; Transcription factors	362 (25)	6.9	16/9
ko04111: Cellular processes; Cell growth and death; Cell cycle-yeast	194 (24)	12.4	7/17
ko04075: Environmental information processing; Signal transduction; Plant hormone signal transduction	225 (23)	10.2	13/10
ko04121: Genetic information processing; Folding, sorting and degradation; Ubiquitin system	585 (22)	3.8	18/4
ko03110: Genetic information processing; Folding, sorting and degradation; Chaperones and folding catalysts	575 (22)	3.8	15/7
ko00230: Metabolism; Nucleotide metabolism; Purine metabolism	430 (21)	4.9	13/8
ko00500: Metabolism; Carbohydrate metabolism; Starch and sucrose metabolism	301 (21)	7.0	12/9
ko04113: Cellular processes; Cell growth and death; Meiosis-yeast	148 (20)	13.5	8/12
ko00040: Metabolism; Carbohydrate metabolism; Pentose and glucuronate interconversions	122 (20)	16.4	7/13
ko00196: Metabolism; Energy metabolism; Photosynthesis-antenna proteins	57 (20)	35.1	0/20
ko00010: Metabolism; Carbohydrate metabolism; Glycolysis/ Gluconeogenesis	361 (18)	5.0	9/9
ko00680: Metabolism; Energy metabolism; Methane metabolism	243 (18)	7.4	9/9
ko01003: Metabolism; Glycan biosynthesis and metabolism; Glycosyltransferases	307 (17)	5.5	8/9
ko04812: Cellular Processes; Cell motility; Cytoskeleton proteins	256 (16)	6.3	0/16
ko00270: Metabolism; Amino acid metabolism; Cysteine and methionine metabolism	235 (16)	6.8	12/4
ko04114: Cellular processes; Cell growth and death; Oocyte meiosis	214 (16)	7.5	8/8

A is the number of total transcripts matched with the pathway; B is the number of total DETs matched with the pathway; Up is the number of up-regulated DETs; Down is the number of down-regulated DETs.

### Validation of data reliability by qRT-PCR

**Fig 6:**
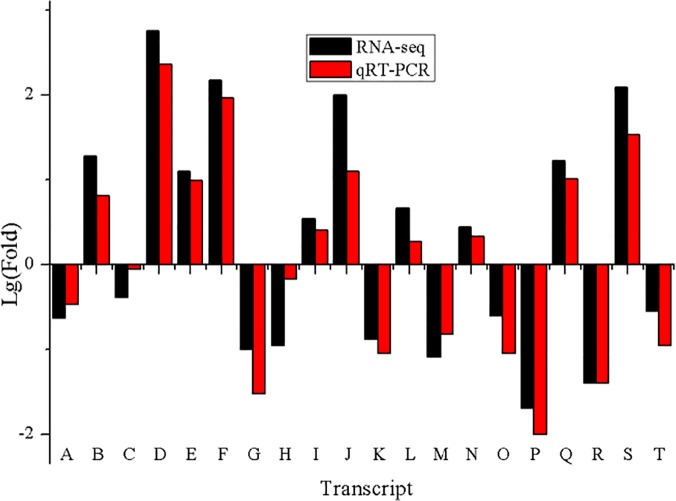
Histogram presentation of the Lg(Fold) of 20 DETs. Black column represents the Lg(fold change (DS/WW)) in transcript expression measured through RNA-seq. Red column is the Lg(fold change (DS/WW)) in transcript expression measured through qRT-PCR. The internal reference gene is glyceraldehyde-3-phosphate dehydrogenase (GAPDH) (comp67082_c0_seq1). A is oxygen-evolving ehancer protein (comp47289_c0_seq1); B is function unknown protein (comp47789_c0_seq1); C is function unknown protein (comp56650_c0_seq1); D is function unknown protein (comp57915_c0_seq1); E is exopolyphosphatase (comp60350_c0_seq1); F is late embryogenesis abundant protein (comp60402_c0_seq1); G is chlorophyll a-b binding protein (comp61248_c0_seq1); H is RSI-1 protein precursor (comp61258_c0_seq1); I is temperature-induced lipocalin (comp62739_c0_seq1); J is protease (comp64242_c0_seq1); K is chlorophyll A/B binding protein (comp63546_c2_seq1); L is heat-shock protein (comp64264_c0_seq1); M is L-ascorbate oxidase (comp65578_c0_seq1); N is function unknown protein (comp67288_c0_seq3); O is light-harvesting complex I protein (comp67308_c0_seq2); P is thaumatin-like protein (comp67881_c0_seq1); Q is BAC insert containing Ma gene (comp68903_c0_seq1); R is dehydration-responsive protein (comp69940_c0_seq1); S is thaumatin-like protein (comp70848_c0_seq2); T is serine/threonine-protein kinase (comp73635_c0_seq2).

To verify the RNA-seq data, we selected 20 DETs for validation using qRT-PCR. Although the fold changes obtained through qRT-PCR displayed some deviations compared with those obtained from high-throughput sequencing (Fig 6), most qRT-PCR-tested transcripts showed a concordant direction of change for both RNA-seq and qRT-PCR. Generally, the qRT-PCR analysis confirmed the transcript expression pattern detected through RNA-seq, indicating that our results were reliable.

### *P*. *mongolica* photosynthesis under drought conditions

**Fig 7:**
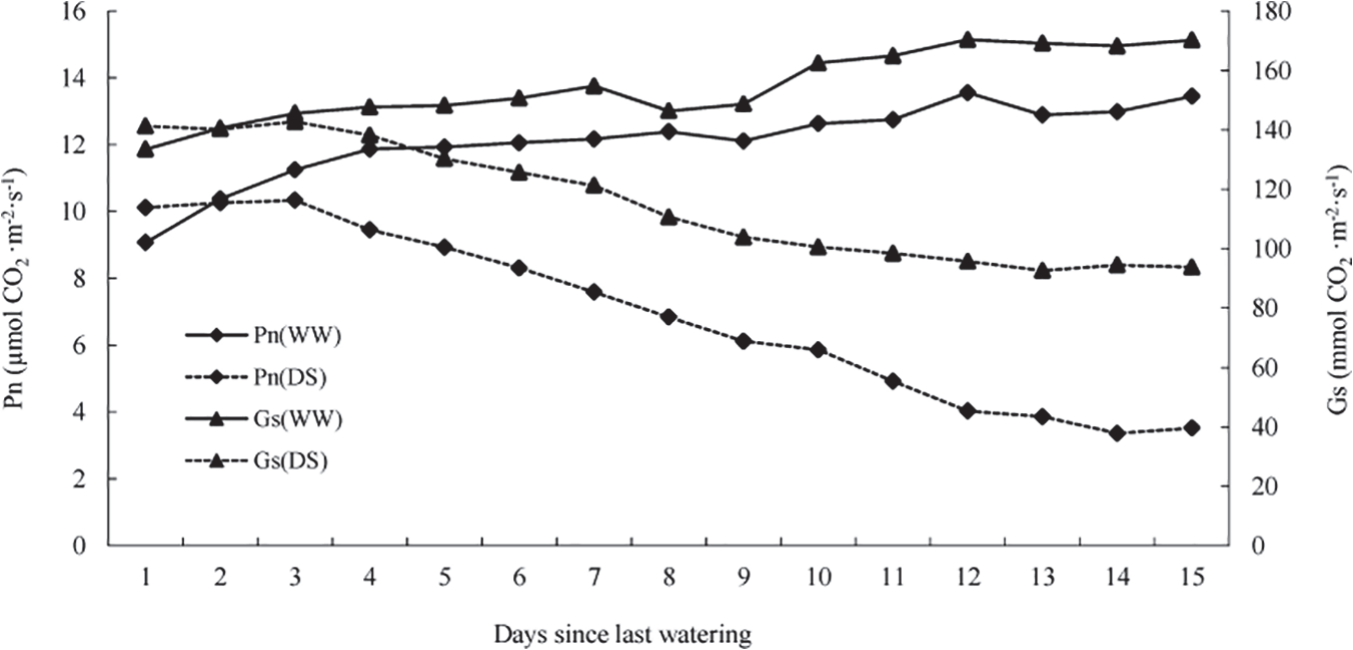
Net photosynthetic rate (Pn) and stomatal conductance (Gs) of well-watered (WW) and drought-stressed (DS) *P*. *mongolica* seedlings.

The Pn and Gs of the WW and DS seedlings are shown in Fig 7. The direction of the change in stomatal conductance is the most important limiting factor for plant photosynthesis [55]. In the present study, when the *P*. *mongolica* seedlings were subjected extended drought conditions, the Pn and Gs showed the same decreasing tendencies observed in plants grown under water deficit conditions (on the 4^th^ day since the last watering); hence, our results support the above conclusion regarding the change in stomatal conductance.

The core metabolism of plants consists of photosynthesis, which provides the energy and basic metabolic substrates for the other major metabolic pathways [56]. Thus, transcripts annotated with GO terms related to the membrane-enclosed lumen, nutrient reservoir activity and macromolecular complexes were down-regulated in the DS treatment (see Fig 5). Among the top 20 KEGG pathways of the DETs, most of the transcripts in the two energy metabolism categories, including photosynthesis proteins (ko00194, S1 Fig) and antenna proteins (ko00196, S2 Fig), were down-regulated, indicating that photosystem I (PSI), photosystem II (PSII) and the light-harvesting chlorophyll protein complex (LHC) also inhibited the photosynthesis of *P*. *mongolica* under the drought condition, similar to the findings of a previous study in wheat [57].

### The balance of plant hormones and DETs in hormone signal transduction

**Fig 8:**
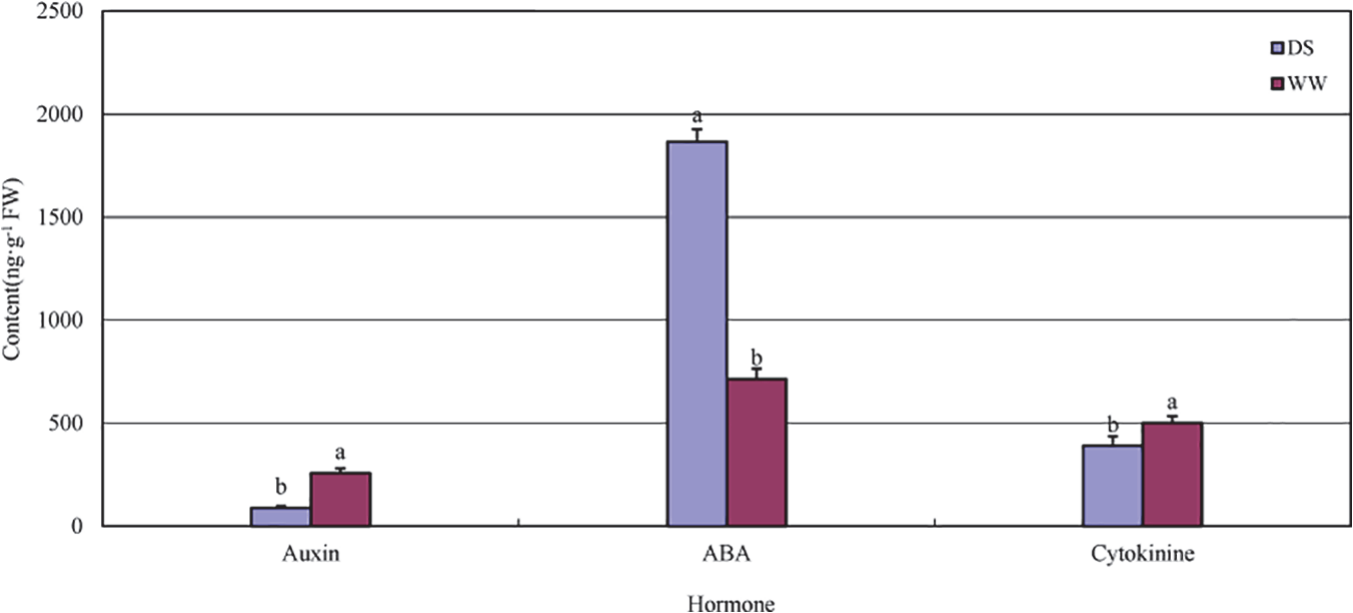
Auxin, abscisic acid (ABA) and cytokinin contents of well-watered (WW) and drought-stressed (DS) *P*. *mongolica* seedlings. Different letters associated with same hormone indicate a significant difference between the WW and DS treatments: Duncan’s multiple range tests at the 5% level (n = 12).

Three types of plant hormones (auxins, abscisic acid (ABA) and cytokinins), were measured to evaluate the hormone balance of *P*. *mongolica* under drought conditions. Compared with the WW seedlings, the ABA content increased significantly under drought conditions, whereas the contents of auxin and cytokinin decreased significantly (Fig 8). The immediate cause of leaf shedding was the increased ABA contents [58]; the DS seedlings shed 3.3 old leaves over the 15-day period on average (see Table 2).

Based on the enzymes reported by Davies [59], the most homologous transcripts involved in plant hormone signal transduction pathways in *P*. *mongolica* were examined in our RNA-seq data (ko04075, S3 Fig). The transcription of type 2C protein phosphate (*PP2C*), which is one of the most important genes involved in ABA signal transduction and is negatively regulated in the plant drought response [60], was up-regulated in the DS treatment. In contrast, the expression levels of *AUX*1, *AUX*/*IAA*, Gretchen Hagen 3 (*GH*3), small auxin up RNA (*SAUR*) and histidine-aspartate phosphorelays (*AHP*) were decreased; these transcripts are involved auxin and cytokinin signal transduction. As a direct result of the changes in these 6 transcripts, the stomata closed, and the enlargement and division of plant cells were inhibited. These results were verified by the lower values obtained for the leaf length, leaf width (Table 2) and Gs (Fig 7) of the DS seedlings. Generally, large leaves use more water. Thus, in water-limited environments, large leaves may be disadvantageous as long as reproductive success is not completely dependent on vegetative biomass [61,62]. Hence, the smaller shoots of *P*. *mongolica* observed during vegetative stages may reduce water use, which may be a factor related to the survival of this shrub in the Gobi Desert.

### The leaf water potential and DETs in the aquaporin gene family

**Fig 9:**
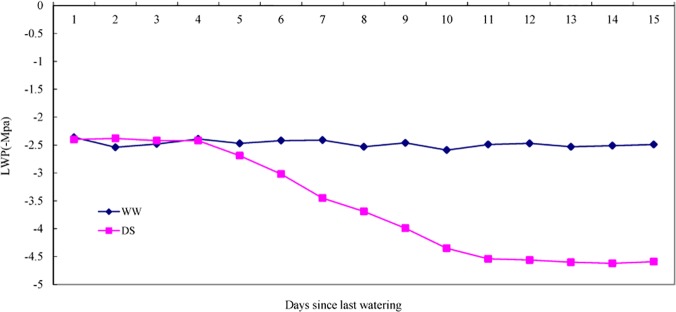
The leaf water potential (LWP) of well-watered (WW) and drought-stressed (DS) *P*. *mongolica* seedlings.

The water potential of *P*. *mongolica* leaves showed significant differences between the WW and DS seedlings (Fig 9). The control seedlings exhibited a stable, higher leaf water potential (LWP). In contrast, the plants grown under water-limited conditions displayed a decreased LWP as the drought strengthened compared with their early drought response, whereas the DS seedlings maintained a stable, lower LWP over the last 5 days. Aquaporins [63] are closely related to the maintenance of the water potential; thus, we investigated the related DETs in the aquaporin gene family.

Aquaporin proteins facilitate osmosis by forming water-specific pores as an alternative to water diffusion through the lipid bilayer, thus increasing the water permeability of the membrane under drought condition [64]. Phylogenetic analysis has revealed that aquaporin genes can be largely divided into at least six different subfamilies [65]: plasma membrane intrinsic proteins (PIPs), tonoplast intrinsic proteins (TIPs), nodulin intrinsic proteins (NIPs), small basic intrinsic proteins (SIPs), hybrid intrinsic proteins (HIPs) and unrecognized X intrinsic proteins (XIPs). Aquaporins influence plant drought resistance, but to the best of our knowledge, no comprehensive analysis of all of the aquaporin genes of a given plant species has been reported thus far.

In total, 42 transcripts belonging to five aquaporin subfamilies (excluding HIPs) were found in our RNA-seq data (S5 Table). We identified 16 possible PIP transcripts through BLAST [26] analysis; only one PIP transcript was significantly down-regulated at the p≤0.01 level, but the ratios of these transcripts were generally lower than 1 (except for some low-abundance (TPM≤10) transcripts). A previous study indicated that decreasing PIP expression reduces cellular water loss under drought conditions [66]. Thus, *P*. *mongolica* may adopt the same strategy to address water deficiency. PIP gene expression is regulated by the abscisic acid (ABA) concentration in plants during drought stress [67], and all of the identified PIPs were down-regulated in the DS treatment, which may be a result of the change in hormone (ABA) signal transduction in *P*. *mongolica* seedlings.

Pou et al. (2013) [66] examined the role of aquaporins in regulating leaf hydraulic conductance in *Vitis vinifera* and found that water stress increased the expression of *Vv*TIP1;1 but decreased the expression of *Vv*TIP2;1. Additionally, the expression of *Vv*TIP2;1 was shown to be highly correlated with stomatal conductance, and the expression of *P*. *mongolica* TIP transcripts observed in the present work is in agreement with this previous study. In addition, the changes in TIP expression in the vacuoles of plant guard cells activate the coupled ion channel, resulting in ion outflow, cell shrinkage and stomatal closure [68]. Cell shrinkage and stomatal closure were also shown to result from hormone signal transduction in *P*. *mongolica* (see S3 Fig), which suggested that TIP transcript expression may be regulated by plant hormone signal transduction.

In addition, NOD-26, SIP1 and most NIPs [69] and SIPs [70] transport small molecules such as glycerol, urea, boric acid and silicon but show poor permeability to water. In the *P*. *mongolica* transcriptome, most of the calculated NIP and SIP ratios ranged form 0.573 to 1.292; the ratios of *PmNIP*9 (comp70470_c0_seq1) and *PmNIP*10 (comp70470_c0_seq2) were 0.352 and 0.354, respectively, but these two transcripts are low-abundance transcripts. In *P*. *mongolica*, the present results showed no obvious changes in NIP and SIP expression under drought conditions.

### Total SOD activities and DETs in the SOD gene family

In total, 177 transcripts involved in antioxidant activity were defined in the RNA-seq data. These defined functions suggested that reactive oxygen species (ROS) were generated as a result of environmental stress, and an effective ROS scavenging and signaling system may exist in *P*. *mongolica* to address drought stress. SOD is a core enzyme in the ROS scavenging system. It is well known that SOD may be divided and grouped into four types of categories: manganese superoxide dismutase (Mn-SOD), copper/zinc superoxide dismutase (Cu/Zn-SOD), iron superoxide dismutase (Fe-SOD) and nickel superoxide dismutase (Ni-SOD); these divisions correspond to the metallic ion in the prosthetic group [71]. Ni-SOD has only been found in microorganisms to date [72]. We observed that total SOD activity in the DS treatment was 129% higher than in the WW treatment. Hence, the question arises of what contributions the three types of SOD (Mn-SOD, Cu/Zn-SOD and Fe-SOD) make to increasing total SOD activity in drought-stressed *P*. *mongolica* seedlings.

**Fig 10:**
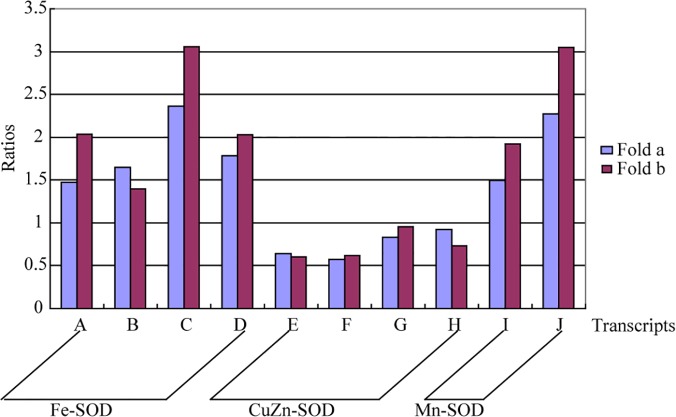
Histogram presentation of the ratios of 10 high-abundance SOD transcripts. Fold a represents the fold change (DS/WW) in transcript expression measured through RNA-seq. Fold b is the fold change (DS/WW) in transcript expression measured through qRT-PCR. A is comp65789_c0_seq1; B is comp65789_c0_seq2; C is comp65789_c0_seq3; D is comp65789_c0_seq4; E is comp61947_c0_seq1; F is comp63868_c0_seq1; G is comp68247_c0_seq1; H is comp70463_c0_seq1; I is comp63735_c0_seq1; J is comp70328_c0_seq1.

In total, 50 transcripts were predicted to show SOD activity, but most of them were low-abundance transcripts. The expression of 10 high-abundance transcripts is presented in Fig 10. The Cu/Zn-SOD ratios were lower than 1, which indicates that drought inhibited the expression of the Cu/Zn-SOD gene in *P*. *mongolica*. However, the expression of the Fe-SOD and Mn-SOD transcripts was up-regulated at this time; in particular, 3 transcripts (A, D and J) were significantly up-regulated in the DS treatment. In addition, we noted that many low-abundance Fe-SOD transcripts were only induced in the DS library (data not shown).

Bhoomika et al. (2013) [73] indicated that the presence and elevated activity of Fe-SOD enhance the activities of Mn-SOD in aluminum-tolerant *Oryza sativa*, and Signorelli et al. (2013) [74] found that drought induces the accumulation of Fe-SOD and Mn-SOD to high levels, but not Cu/Zn-SOD in Lotus (*Lotus corniculatus*). Based on the above two studies and our results, we can deduce that *P*. *mongolica* activates Fe-SOD genes and enhances the expression of Mn-SOD genes to increase total SOD activity under drought stress conditions.

## Conclusions

Mongolian almond (*P*. *momgolica*) is widely established in the Gobi Desert and shows extreme tolerance to drought. To investigate the mechanisms that allow *P*. *mongolica* to maintain growth in extremely arid environments, the response of *P*. *mongolica* seedlings to drought stress was analyzed using morphological, physiological, biochemical and high-throughput sequencing approaches. Compared with the no-stressed control, 3,365 transcripts were differentially expressed in drought-stressed *P*. *momgolica* seedlings, including 1,876 up-regulated transcripts and 1,489 down-regulated transcripts. After being treated with drought stress, the photosynthesis response showed a decreasing tendency, but the changes in the levels of hormones (auxins, cytokinins and abscisic acid) resulted in the closing of stomata and decreased cell enlargement and division; these changes were effective for promoting *P*. *mongolica* survival in the Gobi Desert. Then the aquaporin and superoxide dismutase gene families were analyzed, it is found that all of the plasma membrane intrinsic protein transcripts were down-regulated in the drought-stressed treatment, whereas drought did not affect the expression of nodulin intrinsic protein or small basic intrinsic protein transcripts in *P*. *mongolica* seedlings. In addition, activation of iron superoxide dismutase transcription and enhanced transcription of manganese superoxide dismutase were observed in *P*. *mongolica* to promote tolerance of drought stress. This study identified drought response genes in *P*. *mongolica* seedlings. Our results provide a significant contribution to the understanding of how *P*. *mongolica* responds to drought stress at the transcriptome level, which may help to elucidate the molecular mechanisms associated with the drought response of almond plants.

## Supporting Information

S1 Fig Differentially expressed transcripts among photosynthesis proteins (ko00194). A green box represents a down-regulated transcript in the DS treatment.
(TIF)

S2 Fig Differentially expressed transcripts among photosynthesis antenna proteins (ko00196). A green box represents a down-regulated transcript in the DS treatment.
(TIF)

S3 Fig Differentially expressed transcripts among plant hormone signal transduction pathways (ko04075). A red box represents an up-regulated transcript in the DS treatment. A green box represents a down-regulated transcript in the DS treatment.
(TIF)

S1 Table Primer pairs used for qRT-PCR. (XLSX)

S2 Table Overview of the total transcripts matched with the COG database. (XLSX)

S3 Table Detailed information for the total transcripts matched with the COG database. (XLSX)

S4 Table KEGG classification of the total transcripts. (XLSX)

S5 Table Expression of transcripts from the *P*. *mongolica* aquaporin gene family. Fold^a^: Fold change (DS/WW) in transcript expression measured through RNA-seq. Fold^b^: Fold change (DS/WW) in transcript expression measured through qRT-PCR. “—” indicates not detected in the qRT-PCR analysis.
(XLSX)
