# Supplementary figures and images for: Mongolian Almond (Prunus mongolica Maxim): The Morpho-Physiological, Biochemical and Transcriptomic Response to Drought Stress
Source: PLoS One. 2015 Apr 20;10(4):e0124442. doi: 10.1371/journal.pone.0124442 (PMC4404049; doi:10.1371/journal.pone.0124442)

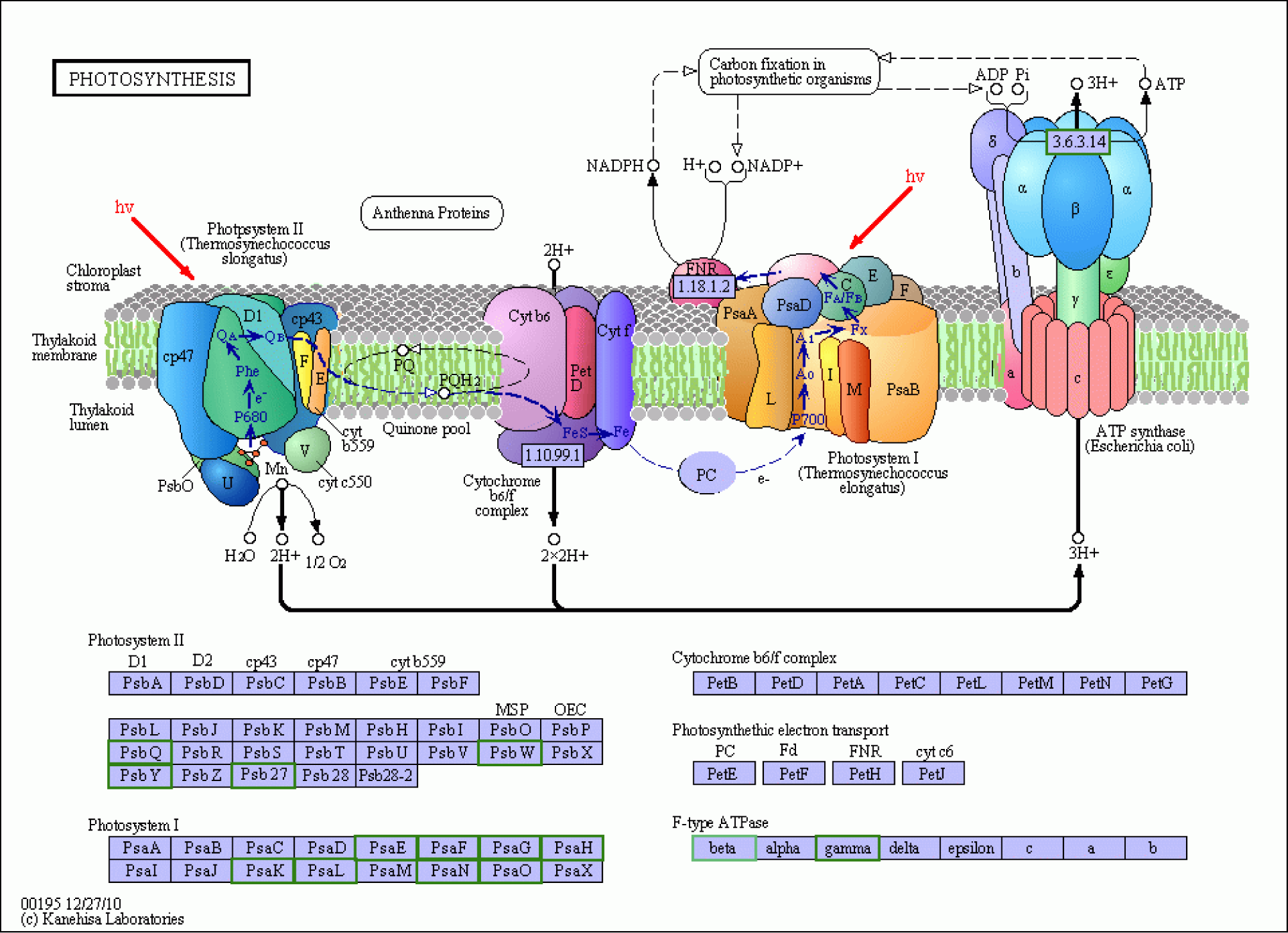

Supplement: S1 Fig — A green box represents a down-regulated transcript in the DS treatment. (TIF) [file pone.0124442.s001.tif]

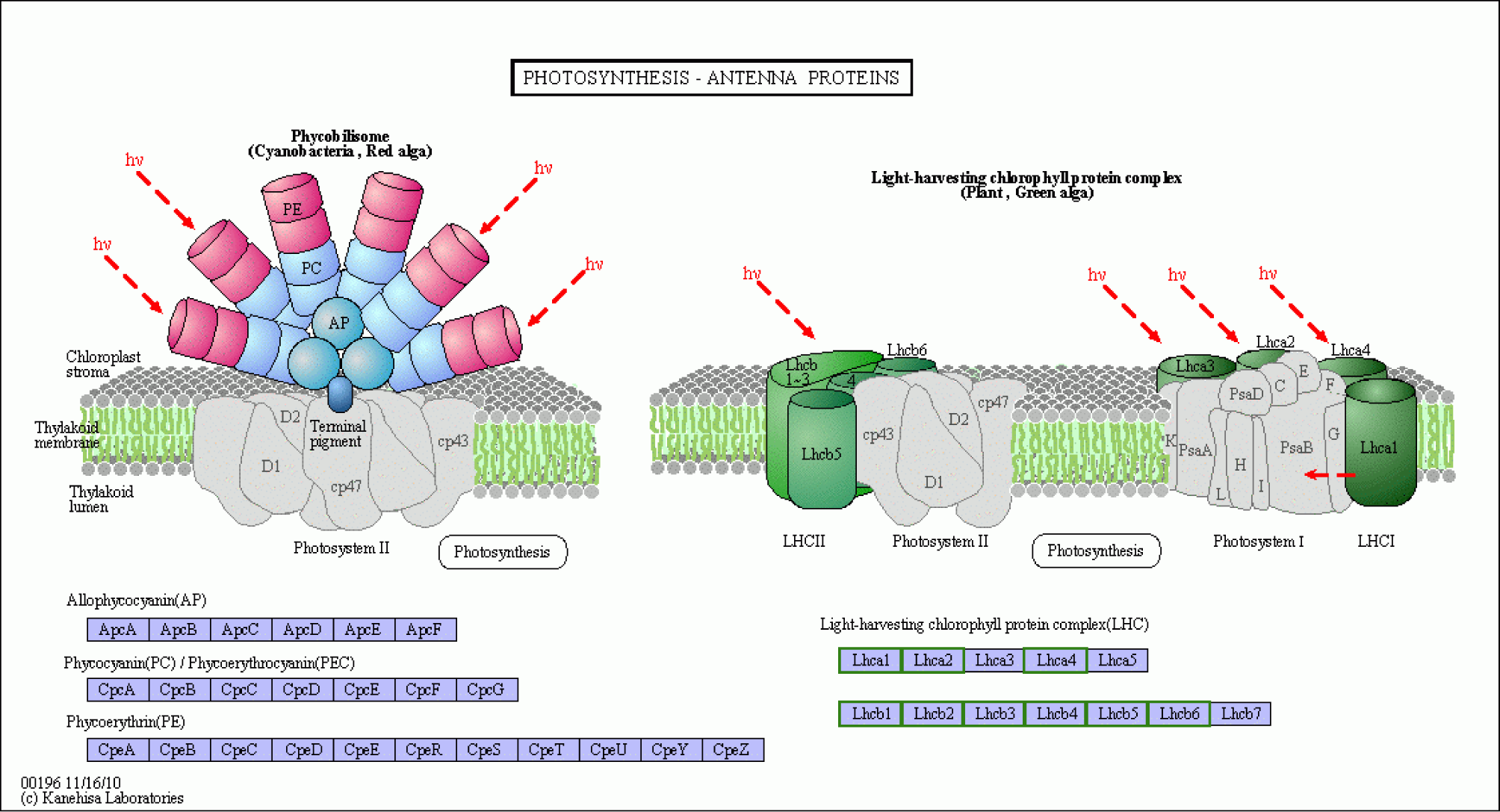

Supplement: S2 Fig — A green box represents a down-regulated transcript in the DS treatment. (TIF) [file pone.0124442.s002.tif]

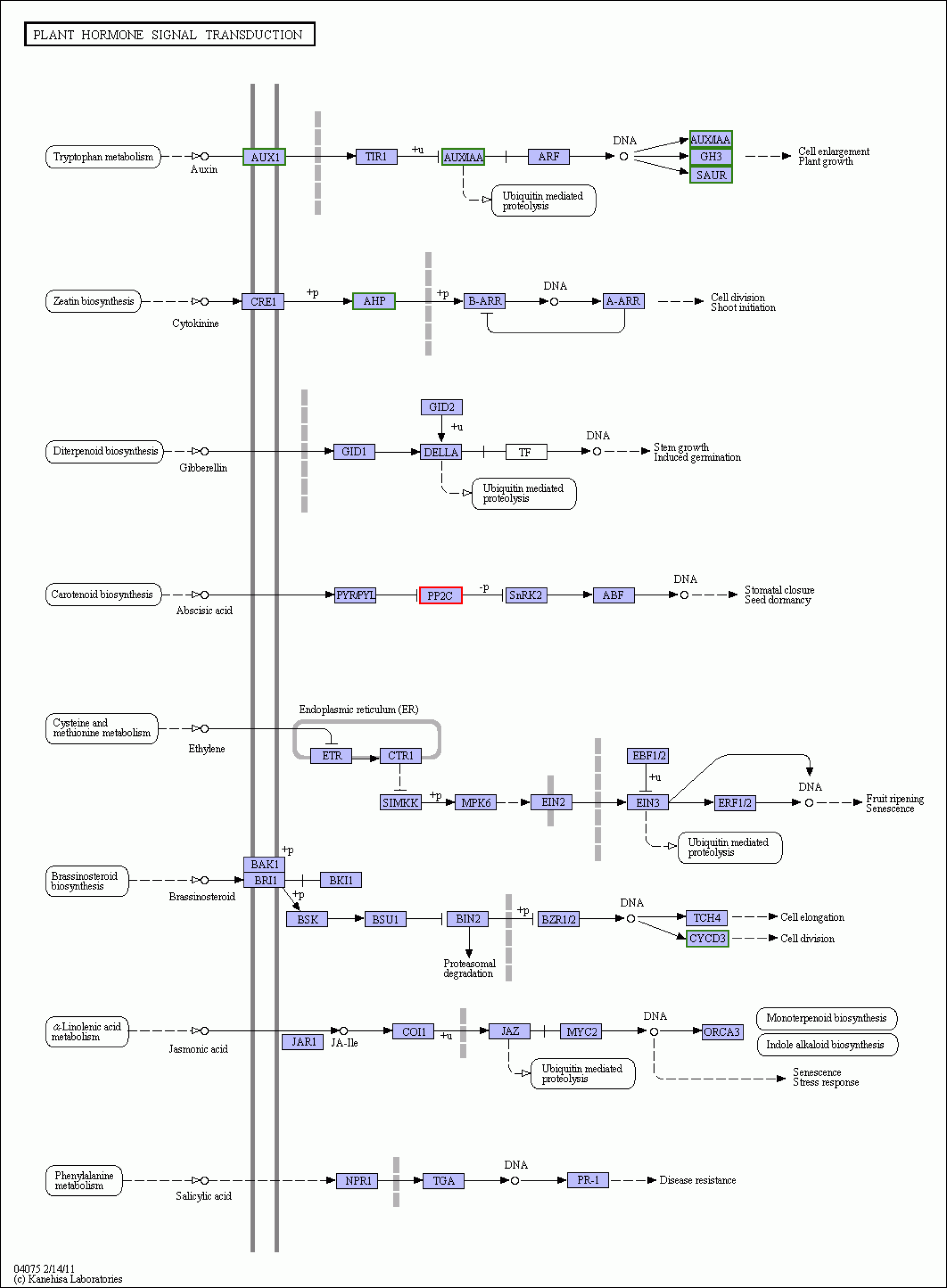

Supplement: S3 Fig — A red box represents an up-regulated transcript in the DS treatment. A green box represents a down-regulated transcript in the DS treatment. (TIF) [file pone.0124442.s003.tif]
